# Supporting Visualization Analysis in Industrial Process Tomography by Using Augmented Reality—A Case Study of an Industrial Microwave Drying System †

**DOI:** 10.3390/s21196515

**Published:** 2021-09-29

**Authors:** Yuchong Zhang, Adel Omrani, Rahul Yadav, Morten Fjeld

**Affiliations:** 1 Division of Interaction Design and Software Engineering, Department of Computer Science and Engineering, Chalmers University of Technology, SE-41296 Gothenburg, Sweden; fjeld@chalmers.se; 2 Institute for Pulsed Power and Microwave Technology (IHM), Karlsruhe Institute of Technology (KIT), 76133 Karlsruhe, Germany; adel.hamzekalaei@kit.edu; 3 Department of Applied Physics, University of Eastern Finland, FI-70210 Kuopio, Finland; rahuly@uef.fi

**Keywords:** industrial process tomography, data processing and visualization, microwave tomography, augmented reality, time-reversal imaging, dyadic Green’s function, multilayered media

## Abstract

Industrial process tomography (IPT) based process control is an advisable approach in industrial heating processes for improving system efficiency and quality. When using it, appropriate dataflow pipelines and visualizations are key for domain users to implement precise data acquisition and analysis. In this article, we propose a complete data processing and visualizing workflow regarding a specific case—microwave tomography (MWT) controlled industrial microwave drying system. Furthermore, we present the up-to-date augmented reality (AR) technique to support the corresponding data visualization and on-site analysis. As a pioneering study of using AR to benefit IPT systems, the proposed AR module provides straightforward and comprehensible visualizations pertaining to the process data to the related users. Inside the dataflow of the case, a time reversal imaging algorithm, a post-imaging segmentation, and a volumetric visualization module are included. For the time reversal algorithm, we exhaustively introduce each step for MWT image reconstruction and then present the simulated results. For the post-imaging segmentation, an automatic tomographic segmentation algorithm is utilized to reveal the significant information contained in the reconstructed images. For volumetric visualization, the 3D generated information is displayed. Finally, the proposed AR system is integrated with the on-going process data, including reconstructed, segmented, and volumetric images, which are used for facilitating interactive on-site data analysis for domain users. The central part of the AR system is implemented by a mobile app that is currently supported on iOS/Android platforms.

## 1. Introduction

### 1.1. Industrial Process Tomography

**Figure 1:**
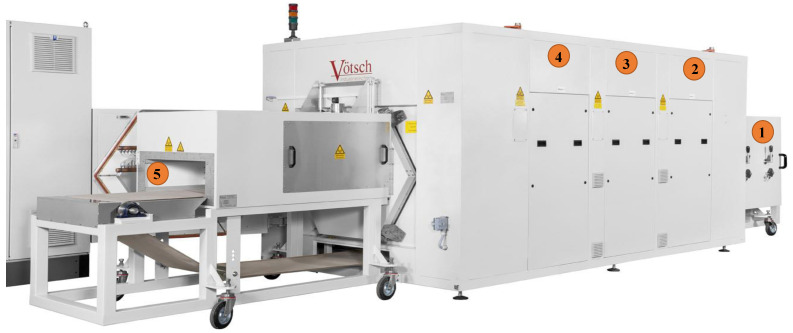
A side view of the HEPHAISTOS microwave oven system. Main modules of the oven are represented by number tags 1, 2, 3, 4, 5. Tag 1 and Tag 5 represent the entrance of the wet foam and exit doors for the dry foam, respectively on the conveyor belt. Tags 2, 3, 4 indicate the three modular heating system with inbuilt hexagonal cavity with high power microwave heating sources and control system block. The MWT system will be integrated near the entrance door. The system is located at KIT, Germany.

Industrial process tomography (IPT), as a non-intrusive and commonly-used imaging technique, has been effectively harnessed in many manufacturing components for inspections, monitoring, product quality control, and safety issues [1,2,3,4,5,6]. IPT underpins and facilitates the extraction of qualitative and quantitative data regarding the related industrial processes, which is usually visualized in various ways for people to understand its nature, measure the critical process characteristics, and implement process control in a complete feedback network [7]. Some typical representatives of IPT, such as microwave tomography (MWT) [8,9,10,11,12,13,14,15], electrical resistance tomography (ERT) [16], and electrical capacitance tomography (ECT) [17] are widely used for industrial purposes such as moisture detection [8,9], crack detection and powder flow in pipes, and flow pattern detection of granules. In our study, we concentrate on a unique industrial microwave drying process [10] which uses precise drying and heating equipment for polymer foams with the aid of MWT, as displayed in Figure 1. The targets of this drying process are firstly heating the object foam placed on the conveyor belt and then detecting the post-drying moisture distribution, by adopting the MWT technique. In this context, it is therefore critical for domain users to comprehend and interpret the process data for intelligent control purposes. Hence, how to develop effective tools or systems which provide people with straightforward and comprehensible visualizations pertaining to the process data becomes a central topic. In this work, we propose a novel and comprehensive solution to support process-critical visualization analyses through the specific case–the MWT-controlled industrial drying processes–by exploiting the interactive augmented reality (AR) technique.

### 1.2. HEPHAISTOS Heating Technology and Microwave Tomography

Drying by microwaves has been widely used, especially in industry, for different applications and purposes. Microwaves can penetrate into the material and provide volumetric heating, in contrast to conventional methods. In microwave drying applications, providing sufficient uniformity of heating distribution is imperative, especially in industrial-scale production [18]. Intelligent control [19] of distributed microwave sources (magnetrons) is a novel idea to increase the efficiency of the drying system, resulting in high-quality processing and reducing the drying time, both of which are highly desirable for the industry. During the drying process, it is only possible to adjust the amplitude and pulse duration of the magnetrons. The decision for controlling these parameters is chosen based on the input information of the moisture location and its level inside the sample.

The microwave drying system which we are presently working with is named HEPHAISTOS (shown in Figure 1). The oven has length and height of 3 m and 1 m, respectively, and is equipped with 18 magnetron sources operating at a frequency of 2.45 GHz. This industrial microwave applicator has a patented hexagonal [20,21] cross-section design delivering high electromagnetic (EM) field homogeneity during the drying process. The system is equipped with a conveyor belt that enables the continuous drying process. Its principal areas of applications are in material processing, for example, thermal curing of fiber composites and drying of porous foams. An MWT system [8] is designed and integrated with the HEPHAISTOS to recover the volumetric information of the moisture location and its level during the drying process of the polymer foam. The power level and pulse duration of the magnetrons will be adjusted based on the input information from the MWT.

### 1.3. Visualization and Augmented Reality

Visualization plays a dominant role in tomography-controlled industrial processes in that it can display the relevant data in an observation-friendly manner, which is then provided to domain users. The information related to the designated process, for example, a 2D graph of the tomographic spectrum or a 3D reconstruction of the voxel data, if collected and visualized instantly in a reasonable way, can help in better understanding of the process among the users [7]. Sophisticated data analysis always demands efficient reproduction of the measurement regarding the imaging process by IPT to uncover the material distribution inside the closed containers where the industrial process is executed [22]. It is therefore imperative to observe and analyze the reconstructed tomographic images which imply the state and status of the process flow, supported with tomograms and graphs, by desirable visualization without interfering the ongoing industrial processes directly [17,23]. Thus, how to get domain users instant and accurate access to various process data by effective visualizations so as to implement real-time data observation and analysis throughout the whole process becomes the main argument of this article.

One of the cutting-edge techniques—augmented reality (AR) (a variation of virtual reality (VR)), in which virtual objects are superimposed in the real world [24]—has been demonstrated and applied in numerous fields due to its capability of providing interactive interfaces of visualized digital content [6,25]. Moreover, AR can provide functional tools that support users undertaking domain-related tasks, especially facilitating them in data visualization and interaction because of its ability to jointly augment the physical space and the user’s perception [7,26]. The adoption of AR in benefiting IPT and its related fields is currently still scarce, resulting in a gap between AR developers and industrial applications [27]. However, some recent research has already pioneered the exploitation of AR to diversify the data visualization and analysis for industrial settings, especially within the context of IPT [6,17,23]. Based on the interactivity and accessibility which AR can provide, we aim to explore the deeper usage of this technique in the IPT domain to support the process data visualization and analysis, by exemplifying a complete case study of the industrial microwave drying process with MWT measurement. The main contributions of this work are as follows:

- Propose an entire data processing and visualizing workflow of the IPT controlled industrial process.
- Pioneer the study of integrating the up-to-date AR technique to support IPT data visualization and on-site analysis for domain users.

This article is organized as follows: In Section 2, the state-of-the-art related work of MWT applications and the exploration of AR in related contexts are introduced. In Section 3, An comprehensive overview of the data processing and visualizing workflow of the case—MWT for microwave drying is presented, including the engaged equipment and materials, of which the time reversal imaging algorithm, the post-imaging segmentation, and the volumetric visualization. The conceptualization and the specification of how the AR approach can support effective data visualization and analysis are given in Section 4. In Section 5, the results of the case study are presented and discussed. The concluding remarks and future work are given in Section 6.

## 2. Related Work

### 2.1. Microwave Tomography for Industrial Process Applications

A comprehensive review on applications of the MWT in the process industry is listed in [28]. Microwave sensing and imaging methods for application in multi-phase flow monitoring and metering in the oil and gas industry are given in [29,30]. In the food industry, MWT has been widely applied for inspection and quality control [31,32,33]. Sensing of low permittivity materials, such as gaseous substances or foam with high air content, with MWT is reported in [34]. Moreover, the authors have reported their contribution in [9,35,36,37] for the present application of MWT in estimating the moisture content in polymer foam.

### 2.2. Visualization and Augmented Reality

Under the circumstances that research into using AR to benefit IPT related fields was insufficient, over the last two decades researchers have been investigating how to apply AR to provide novel and effective visualization approaches for tomographic process data. In 2001, Mann et al. [38] developed an AR application to capture and visualize the dynamics of the mixture in stirred chemical reactors operated by ERT. Recently, Nowak et al. [17] benchmarked a new AR solution to visualize industrial tomography data and further enable collaborative in-situ analysis, by making use of the up-to-date Microsoft HoloLens 2 headset (https://www.microsoft.com/en-us/hololens, 15 August 2021). More interestingly, they formulated deeper research to investigate a more advanced prototype for underpinning complex data visualization and analysis in entire 3D surroundings within the same IPT context [23]. On the premises that some research has opened the horizon of adopting cutting-edge AR to generate effective visualizations for IPT data, we have proposed a novel AR framework for better volumetric visualization and onsite analysis for MWT aided processes [6]. Based on this previous work, this study explores more efficient AR methodologies for domain users by replenishing a complete data processing and visualizing workflow as well as improving the central AR application.

## 3. Data Processing and Visualizing Workflow

The case we deal with in this article is a unique microwave drying process for polymer materials with the aid of MWT measurement. To observe and monitor the whole process, it is critical to unveil the intact dataflow with regard to every phase, and displaying it in a visualization-friendly manner. In this part, we introduce the proposed data processing and visualizing pipeline of our case, consisting of three modules: Time reversal imaging, post-imaging segmentation, and volumetric visualization.

### 3.1. Practical Challenges

To design and integrate an MWT system into an industrial drying system the following practical guidelines should be taken into account. First, the unwanted EM leakage power coming from the entrance aperture can take down the MWT system, and therefore has to be blocked. In this regard, many types of antennas which are already employed for microwave imaging purposes cannot be used in this application. Here, the X-band frequency range for the MWT is chosen, and the WR-90 open-waveguide is employed to act simultaneously as an antenna and microwave high pass filter. Second, fast data acquisition is essential to enable real-time monitoring and providing rapid input information for the control unit. Hence, a high number of antennas cannot be chosen to develop the MWT sensor and more so applying iterative-based optimization techniques are challenging. Third, the imaging modality should be compatible facing different conditions like low or high-contrast media. Consequently, the developed microwave imaging should be compatible with the low number of antennas and be able to perform under the mentioned conditions.

### 3.2. Microwave Tomography: Time-Reversal Imaging Algorithm

**Figure 2:**
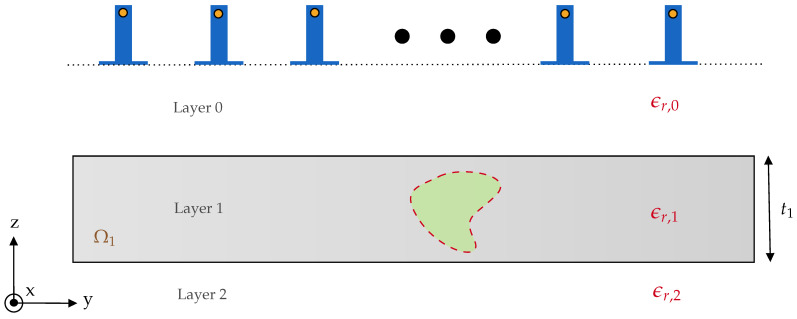
2D schematic of the MWT setup with open-ended waveguide antennas. In the present study, layer 0 and layer 2 represent free-space (air), and layer 1 represents the polymer foam. The region represented by the green color indicates the moisture wet-spot inside the foam.

Here, we use the qualitative time-reversal(TR) imaging for detecting the moisture location inside the polymer foam. The super-resolution characteristic of TR imaging facilitates the detection of the targets even in rich clutter media. The imaging set-up for a fixed cross-section (i.e., y×z), is shown in Figure 2 where layer 0 is the free-space, layer 1 represents the polymer foam with thickness $t_{1}$, and layer 2 is either free-space or a perfect electric conductor (PEC). The multistatic antenna array with *N* element is fixed and the distance of the antenna to the top of the polymer foam is $t_{0}$, and the center to center distance between two adjacent antennas is *d*. In free space, the relative dielectric constant is denoted as $\epsilon_{r,0}$ whereas the relative dielectric constant of the layer 1 and layer 2 are set to $\epsilon_{r,1}$ and $\epsilon_{r,2}$, respectively. In the next section, the multistatic data matrix (MDM) for the electric field will be built, and an asymptotic expression for the Green’s function of the multilayer media will be obtained and used in TR imaging. Here, the main interest is in imaging a subsurface target and extracting a representative 3D model for AR visualization and not determining the target material.

#### 3.2.1. Scattering Model and Time Reversal Imaging

Consider an active array of *N* tranceivers from which an N×N MDM is constructed. Each element of the MDM matrix represents the received scattered field by *i*th (i=1,2,⋯,N) antenna when *j*th (j=1,2,⋯N) antenna is in the transmitting mode due to the inhomogeneities in the region of the interest (ROI) can be expressed [39,40,41]

(1) $$\begin{array}{c} {\vec{E}}_{\mathrm{sct}}^{\left(1\right)}\left({\vec{\rho }}_{r_{i}},{\vec{\rho }}_{t_{j}}\right)=i\omega \mu_{0}\int_{\Omega_{1}}{\bar{\bar{G}}}_{\mathrm{eb}}^{\left(01\right)}\left({\vec{\rho }}_{r_{i}},{\vec{\rho }}^{\prime }\right)\;\cdot \;O\left({\vec{\rho }}^{\prime }\right){\vec{E}}_{\mathrm{tot}}^{\left(1\right)}\left({\vec{\rho }}^{\prime }\right)d{\vec{\rho }}^{\prime }. \end{array}$$

Here, ${\vec{E}}_{\mathrm{tot}}^{\left(1\right)}$ denotes the total electric field in layer 1 and ${\vec{E}}_{\mathrm{sct}}^{\left(1\right)}$ is the scattered field received by the *i*th antennas. $O\left(\rho^{\prime }\right)=-i\omega \epsilon_{0}\left(\epsilon_{\Omega_{1}}-\epsilon_{r,1}\right)$ is the object function where $\epsilon_{\Omega }$ denotes the target relative dielectric constants in the domain $\Omega_{1}$, and $\mu_{0}$ and $\epsilon_{0}$ denote free-space permeability and permittivity, respectively. Here, time convention of $e^{-i\omega t}$ is assumed and suppressed where ω is the angular frequency. In (1), the vector ${\vec{\rho }}_{r}$ and ${\vec{\rho }}_{t}$ represent source and observation points while ${\vec{\rho }}^{\prime }=\left(y^{\prime },z^{\prime }\right)$ is the location of the pixel point in the ROI. ${\bar{\bar{G}}}_{\mathrm{eb}}^{\left(01\right)}$ is the background (multilayer media without any inhomogeneities) dyadic Green’s function (DGF). The superscript (01) denotes that the source point is located in layer 1 and the observation point is in layer 0. It should be noted that the analytical equations are written for the electric fields, however, a de-embedding procedure will be applied later to convert the measured S-parameters to the electric field.

A multistatic model for the received scattered field that describes each element of the MDM matrix can be obtained and written as

(2) $$E_{\mathrm{sct}}^{\left(1\right)}\left({\vec{\rho }}_{r_{i}},{\vec{\rho }}_{t_{j}}\right)=i\omega \mu_{0}\int_{\Omega_{1}}G_{\mathrm{eb}}^{xx}\left({\vec{\rho }}_{r_{i}},{\vec{\rho }}^{\prime }\right)O\left({\vec{\rho }}^{\prime }\right)G_{\mathrm{eb}}^{xx}\left({\vec{\rho }}^{\prime },{\vec{\rho }}_{t_{j}}\right)d{\vec{\rho }}^{\prime }.$$

In deriving (2) from (1) the symmetry property of the DGF is used, and furthermore, it is assumed that both transmitting and receiving antennas are *x*-polarized, so the xx term of the DGF is employed. The MDM can be expressed in the compact form in the angular frequency domain ω as

(3) $$\begin{array}{c} K\left(\omega \right)=i\omega \mu_{0}\int_{\Omega_{1}}O\left({\vec{\rho }}^{\prime }\right)g_{b\rho_{i}}\left({\vec{\rho }}^{\prime },\omega \right)g_{b\rho_{j}}^{{}^{⊤}}\left({\vec{\rho }}^{\prime },\omega \right)d{\vec{\rho }}^{\prime }, \end{array}$$

where $g_{b\rho }={\left[G_{eb}^{xx}\left(\rho ,\rho_{1}\right),G_{eb}^{xx}\left(\rho ,\rho_{2}\right),...,G_{eb}^{xx}\left(\rho ,\rho_{N}\right)\right]}_{N\times 1}^{{}^{⊤}}$ is the frequency-domain steering vectors of the xx components of the background DGF and ${\left(\;\cdot \;\right)}^{⊤}$ is the transpose operator. Decomposition of the time-reversal operator (DORT) can be applied to selectively focus on the inhomogeneities in the media using singular value decomposition (or SVD) of the MDM matrix [42]. The SVD’s of the matrix K(ω) is expressed in terms of the eigenvalues via the equation $K\left(\omega \right)=U\left(\omega \right)\Lambda \left(\omega \right)V^{†}\left(\omega \right)$, where Λ$\left(diag(\Lambda )=[\nu 1,\nu 2,\cdots ,\nu N]\right)$ is a real diagonal matrix consisting of eigenvalues, while *U*$\left(u_{i}\left(\omega \right),i=1,2,\cdots ,N\right)$ and *V*$\left(v_{i}\left(\omega \right),i=1,2,\cdots ,N\right)$ are the left and right matrices consisting of normalized eigenvectors [42,43] of time reversal operator (TRO).

For focusing on the *p*th scatterer, we employ $e_{p}\left(\omega \right)=\lambda_{p}\left(\omega \right)u_{p}\left(\omega \right)$ as a new excitation for the transmitting antennas and calculate the propagated fields using the xx component of the background DGF. Here, $\lambda_{p}$ is the *p*th eigenvalue and $u_{p}$ is the *p*th left singular vector. As a result the location of *p*th scatterer is synthetically obtained by the following imaging function

(4) $$\begin{array}{c} D_{p}\left(\rho \right)=\int_{\omega }e_{p}^{T}\left(\omega \right)g_{b\rho }\left(\rho ,\omega \right)d\omega , \end{array}$$

where ρ is any arbitrary point in the ROI.

#### 3.2.2. TR-DORT Simulation Results

In this section, TR-DORT imaging simulation results are demonstrated using the derived asymptotic Green’s function for different moisture scenarios. To generate the synthetic electric-field data, the 3D scenario of the MWT schematic shown in Figure 2 is developed in the commercial software CST Studio Suite with time-domain solver. In the 3D setup, the foam with dimension $\Omega_{\mathrm{foam}}=\left[-25,\;25\right]\times \left[-15,\;15\right]\times \left[-4,\;4\right]$ is chosen and the open-ended waveguide antennas is placed in free space at a distance of 16cm from top of the foam. The 7×7 MDM matrix is extracted for the bandwidth of 4GHz with the center frequency of 10GHz (X-band range) and the number of frequency is $N_{f}=1001$. It should be noted that the response of the antennas is removed from the measured S-parameter using a calibration proposed in [44,45]. In this study, the dielectric constant of the dry polymer foam is assumed to be known and is $\epsilon_{r,2}=1.16-0.01j$. The wet-spots are modelled by a spherical shape with the defined dielectric constant corresponding to the different moisture levels. The correlation between the dielectric constant and the wet-basis moisture levels is measured by cavity-perturbation method as well as transmission line technique at 2.45 GHz [46]. For this characterization, the polymer foam density is $28\frac{\mathrm{kg}}{m^{3}}$.

**Figure 3:**
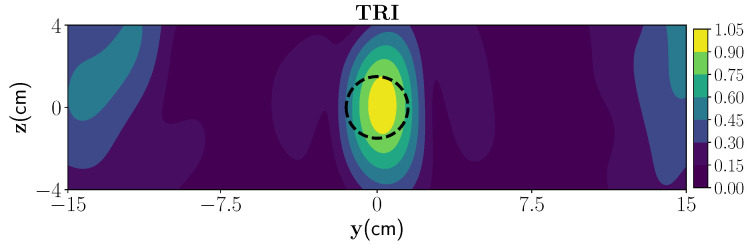
Reconstruction of one wet-spot moisture case with TR-DORT where the true location is marked by black dash lines. The moisture area is represented by the yellow color and the dark blue color represents the dry part.

**Figure 4:**
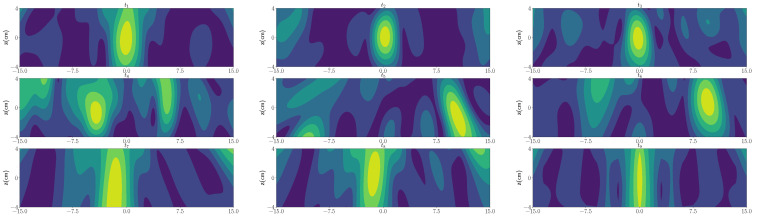
The order of the timestamp-marked TR images: **top left**: $t_{1}$; **top middle**: $t_{2}$; **top right**: $t_{3}$; **middle left**: $t_{4}$; **middle middle**: $t_{5}$; **middle right**: $t_{6}$; **bottom left**: $t_{7}$; **bottom left**: $t_{8}$; **bottom right**: $t_{9}$.

In the first scenario, we have considered one wet-spot in the polymer foam with a radius of 1.5 cm located at the center of the polymer foam, i.e., (0cm,0cm in yz plane with 30% moisture level and rest of the foam is considered dry). Figure 3 shows the reconstruction image associated with the first strong eigenvalue, and employing the derived Green’s function. As can be seen, using TR-DORT, the image domain is decomposed into the wet-spot location and dry parts. Further, we have considered multiple wet-spots with spherical shape (and same moisture levels) located at different positions in certain length of the foam. For this case, the reconstruction using the TR algorithm are performed at different time-stamps and are shown in Figure 4.

### 3.3. Post-Imaging Segmentation

After the designated drying process, under most circumstances there will be some regions of the polymer foam not being completely dry. The difference between dry and non-dry areas is represented by allocating different colors in the reconstructed MWT images, which entail different moisture levels retained in the polymer foams. As shown in Figure 4, the bright yellow parts stand for high moisture while the dark blue parts stand for low moisture. The colors become more yellow when there is more moisture. There exists significant implication for domain users to precisely assess the moisture distribution of the material so as to gauge the success of the conducted drying process.

**Figure 5:**
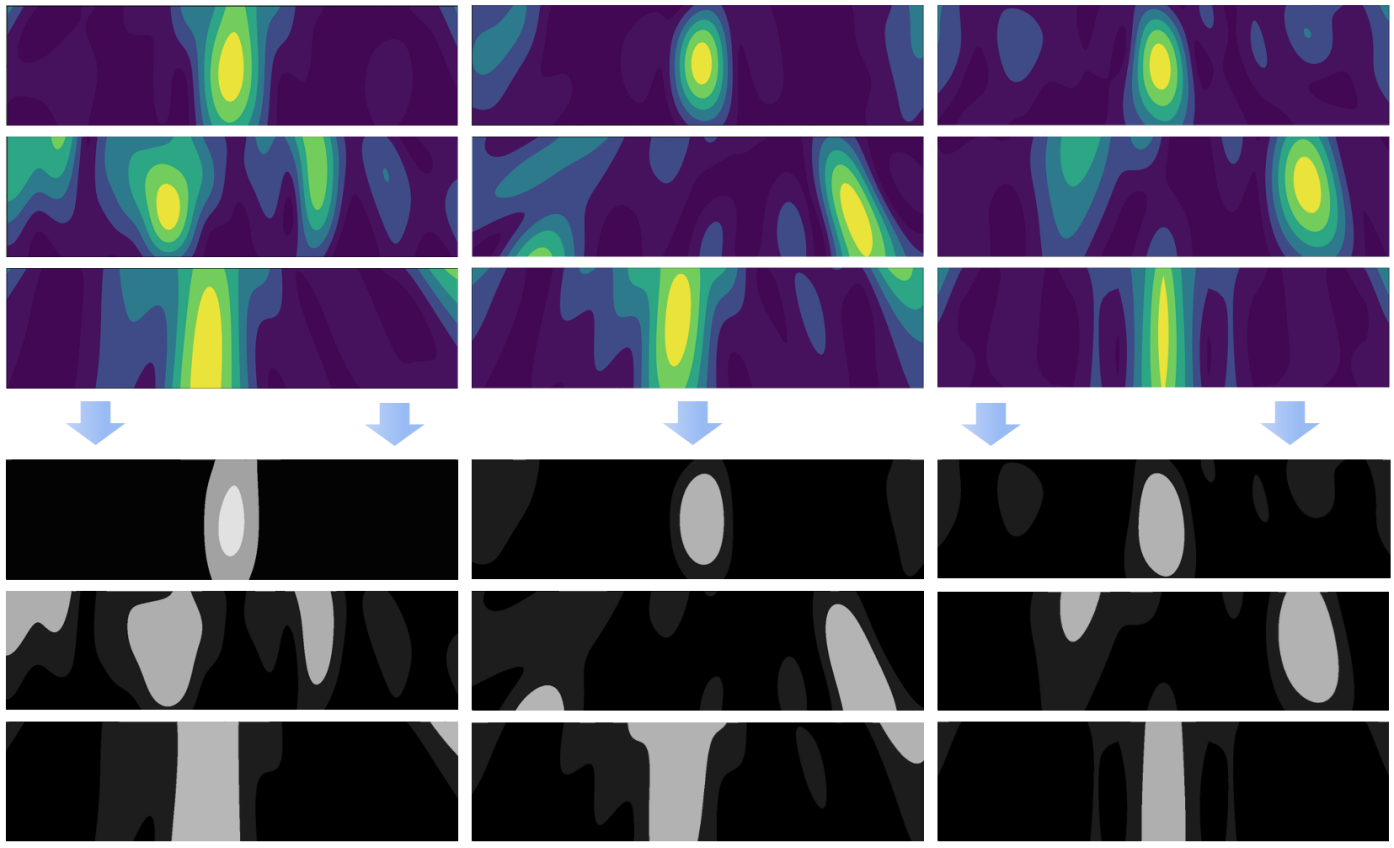
The nine timestamp-marked TR MWT images after reconstruction from the process with their corresponding segmented results. The order of the timestamp-marked images are same as given in Figure 4. In the segmented results, the light grey areas are the visualized parts, which represent high moisture ares of the polymer foam used for drying.

To comprehensively help people to understand the outputted results, as well as to increase the accountability regarding the visualization, we deployed an entirely automatic segmentation method to visualize the high moisture areas on the reconstructed MWT images. Following our previously published work [12], we harness the MWT Segmentation based on K-means (MWTS-KM) segmentation algorithm to highlight the desired high moisture areas of the post-dried polymer foams on the reconstructed MWT images engaged in our study. As shown in Figure 5, we implement the segmentation on the corresponding nine MWT images originating from different timestamps of the drying process. The high moisture areas are displayed with light grey colors. Hence, the domain users are able to observe the segmented results to either conduct further visualization analysis or evaluate the success of the process.

### 3.4. Volumetric Visualization

**Figure 6:**
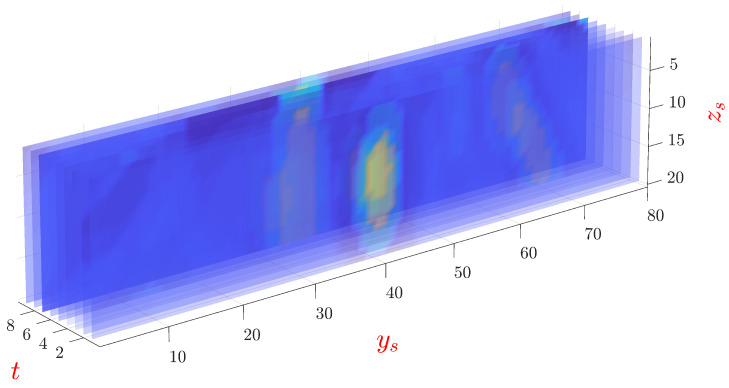
Volumetric visualization of the moisture distribution in the *y*–*z* plane as estimated by the MWT at different time slots. The term $y_{s}$ and $z_{s}$ denotes the pixels in the *y* and *z* directions, respectively. The dominant wet-spots are represented in yellow and the dry part is represented as blue.

For volumetric visualization of the moisture in the foam, the reconstructed moisture in the cross-section of the foam from t=1,2,⋯9 is used. Following the post-imaging segmentation process, the processed TR reconstructed images at different time slots are stacked to represent the three-dimensional information of the moisture, as shown in Figure 6. The dominant wet-spots are represented by the color yellow whereas the dry part is represented by the blue color. The *y*–*z* plane is discretized into 80×20 pixels for simplified rendering and real-time display.

## 4. Augmented Reality for Visualization

**Figure 7:**
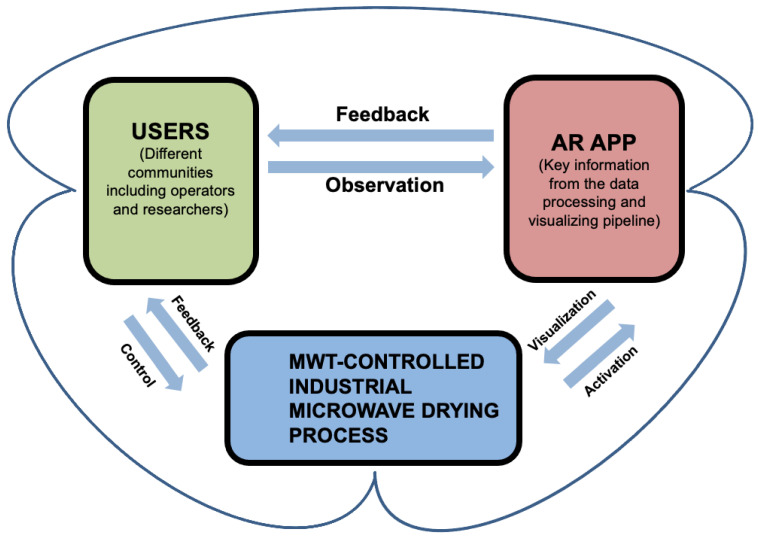
The conceptualization framework of the proposed AR system. MWT-controlled industrial microwave drying process block: This covers the whole running procedure of the process, providing data and elements used for analysis. Users block: This represents the people involved in this context. For instance, the operators who control and run the process and the researchers who observe and analyze the process are the main composition of this part. AR App block: This entails the AR implementation, which was a mobile AR App developed on iOS/Android platform at the initial stage. The App allows different communities of people to get access to the key information from the proposed data processing, visualize workflow interactively, and conduct the onsite analysis collaboratively [6].

To better support the onsite analysis related to the informative data processing and visualizing workflow, we present a novel AR framework which enhances the interactivity and immediacy of data analysis. The proposed system has three main components, as displayed in Figure 7.

- MWT-controlled industrial microwave drying process: A unique heating and drying process operated in a confined chamber with sophisticated industrial settings. In our study, the target is a microwave drying process for polymer foams undergone by the precise HEPHAISTOS microwave oven system shown in Figure 1.
- Users: Operators who control and run the drying equipment, or researchers who take onsite observations and collect data for further analysis.
- AR App: The core part of our proposed system. As a preliminary stage, the application is manifested as a mobile App run in iOS/Android mobile devices, and used for interactive and collaborative volumetric visualization and analysis. In comparison with our previous work [6], a multiple floating interface provided and overlaid in a real environment and containing information from our proposed data workflow is the main component of the application.

**Figure 8:**
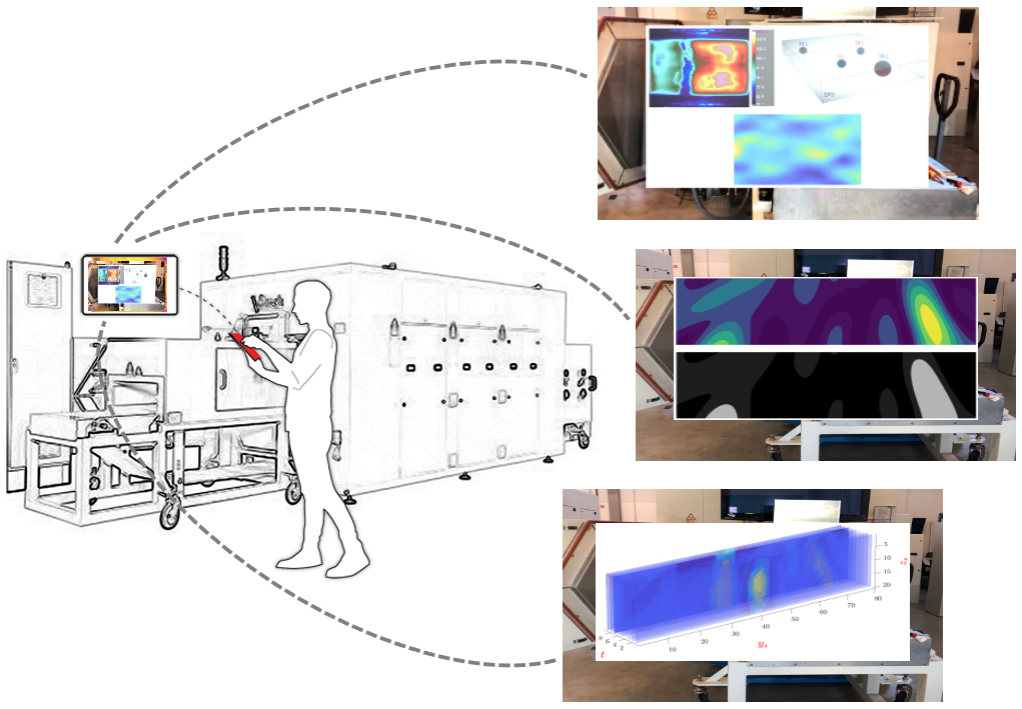
The realization of the proposed AR system. Users hold the handheld smartphones/tablets to run the AR App alongside the industrial setting. After the App activation, users can then observe the visualizations displayed on the AR interfaces. At present, the App is supported via iOS/Android mobile devices. Users are empowered to switch to different visualizations regarding different phases of the process. The visualizations shown on the interfaces are multimodal, including the infrared image showing the condition of the process and the 3D/2D reconstruction of the MWT images (the upper left small figure on the floating interface in the upper right subfigure); the reconstructed MWT images with their segmented results to show the high moisture areas (the middle right subfigure); the volumetric visualization (the bottom right subfigure) [6].

The principal part of the initiated AR system is a mobile App developed to visualize the necessary information revealed from the aforementioned data processing and visualizing pipeline, while it supports interactive and collaborative onsite data analysis. This App serves as an intermediary tool between the users and the precise experimental equipment used for displacing the visualized information regarding the ongoing process. The users, for example, operators, can interact with the visualizations projected on the AR interface, with the mobility of merely holding a mobile device alongside the bulky equipment. As Figure 8 illustrates, people from different communities, such as practical operators or academic researchers, have the capacity to interactively and instantly observe the information related to the process by simply activating the App without initiating other facilities. The App was developed via Unity and Vuforia engines, aiming at the present stage for iOS/Android portable devices such as smartphones and tablets. By pointing the camera of the devices towards the precise equipment (the HEPHAISTOS system) used for performing drying and heating, the App will then be activated, displaying a floating interface with the necessary information (figures, images, etc) virtually superimposed. Compared to our previous work [6], the application itself has been ameliorated to not only a single interface but on-site multiple interfaces containing various visualizations obtained from the data pipeline. Users are able to switch to the visualizations they require for the desired analyses. As shown in Figure 8, some different floating interfaces incorporating different modalities of visualizations are presented and supported by switching after activating the App. The mobility of the AR interface provides users with ongoing visualizations as they move around the equipment that runs the designated process.

## 5. Discussion

In the article, we propose a comprehensive and informative data processing and visualizing workflow regarding IPT related fields by concentrating on a specific case–an MWT controlled industrial microwave drying system. We elicit the satisfactory time reversal imaging algorithm, the automated post-imaging segmentation, and the volumetric visualization which characterize the whole process of our case. Furthermore, we present a novel AR framework to support the visualization analysis by incorporating the necessary information used for practical needs. In this part, we narrate the insights gained as well as the existing limitations of our methodology.

### 5.1. Insights

The first concern of our study is how to present a complete data processing pipeline with informative visualizations, while these modalities are incorporated by the second focus–a preliminary AR framework to facilitate interactive and collaborative onsite data analysis regarding volumetric visualization in IPT systems. As a pioneer study in this related context, our proposed AR system opens the new horizon of deploying this interactive technique into IPT, boosting the complicated data processing and visualizing with an MWT-controlled microwave drying process. Based on our study, we present the following findings:

**Interactivity.** The most prominent advantage of this system is that the information visualization by the AR interface is completely interactive. By using the AR App after activation, a user can easily get instant access to the visualized data by observing the interface which incorporates the information needed for decision-making. In addition, users are offered the option of focusing on different visualizations from among the different modalities of data we have projected into the virtual interface. Our AR system provides a new perspective of interactive data analysis to benefit IPT related users.

**Mobility.** Another strength of this system is its mobility. Generally, controlling and supervising an IPT related process requires implementation in specific and precise equipment and scientific laboratories, such as the drying apparatus and the HEPHAISTOS laboratory as mentioned before. Researchers who observe the data and analyze the ongoing process invariably conduct this work on dedicated computers located at different places. However, with this system they are empowered to hold a portable mobile device (a smartphone or tablet) with a running App to closely implement the data analysis beside the precise equipment. The mobility of our system greatly reduces the time cost while improving the working efficiency.

**Information richness.** The AR interface is used for displaying the significant information needed for boosting the decision-making of the industrial process. In our example, we showcase the visualized data characterizing the specifications of the process. Specifically, we report the first AR application of bringing a complete data processing and visualizing workflow related to complex IPT process which contains the key information used for further analysis. For instance, in our MWT–controlled microwave drying process, a generated MWT image by time-reversal technique is able to show the precise moisture distribution, while the up-to-date volumetric visualization has the capability to reveal the parameters of the drying material and the operating situation of the equipment.

**Mutual collaboration.** Our system enables in-situ collaboration by providing a number of virtual AR interfaces comprising different information, including all the sophisticated visualizations emerging from the workflow (Figure 8). Under this setting, users from different communities, such as hands-on operators, academic researchers, and engineers, can independently conduct visualization analysis by observing the corresponding visualizations, and interact with each other on common problems as well. This shared mode breaks the gap of different communities working separately but then integrates them into a complete human-in-the-loop working pattern. Through our system, users concentrating on distinct targets originally can jointly interact and discuss at the same location, simultaneously having the access to the same interface containing visualizations.

### 5.2. Limitations

While we have listed some superiorities of the our proposed solutions, we must also acknowledge there still exist some deficiencies in this preliminary AR framework supporting the visualization of data workflow. Foremost, the dataflow itself does not include all the necessary information related to the MWT-controlled process. For instance, only one modality of volumetric visualization is discussed in our study, whereas involving more formats of data presentations would be more advantageous. Moreover, although we have set a precedent for bringing the AR technique to benefit IPT especially in complex visualization analysis, we have not yet evaluated our system through a systematic user study to gain constructive and critical feedback. Obtaining early-stage findings has not been sufficient for formulating a reliable and robust AR system which should be deemed as a supportive tool in industrial settings. Only those prototypes which are established and improved based on proper usability testing can be the real-time applications at this stage. That is, a comprehensive experiment of user aspects should be mandatory for a constructive human-in-the-loop system, and is not included in this study at current stage.

In addition, our preliminary AR system is embodied as a mobile App on portable smartphones or tablets. This setting can provide the fundamental AR experience for targeted users, but it limits the AR interface to small, bounded screens. Generally, a functional AR application elicits high interactivity and immersivity to users due to its property to put the virtual elements in front of people. The formulation of AR in handheld mobile devices to some extent hinders the essential advantages of AR, which should be tackled by introducing more advanced headsets that can make AR scenes more interactive and immersive.

## 6. Conclusions and Future Work

In this article, we first propose a complete data processing and visualizing workflow in IPT system by using the MWT-controlled microwave drying case, then we present a preliminary AR system in the same context to support complex visualization analysis. The data pipeline contains an up-to-date time reversal imaging algorithm for reconstructing MWT images, a post-segmentation part which automatically visualizes the desired areas on MWT images, and a straightforward volumetric visualization part. The overall realization of the reported AR framework is conducted by a mobile AR App run on smartphones/tablets, aiding our specific MWT controlled microwave drying process. We successfully prove that this novel system brings new conceptualization in this domain, by offering interactivity and mobility on users’ in-situ data analysis and visualizations. Moreover, we emphasize that our AR system has been improved compared to the previous version by eliciting multiple switchable floating interfaces which incorporate multimodal visualizations of the designated process. Thus, the significant increase of the information richness in this interdisciplinary area is generated. The emergence of this system facilitates mutual collaboration by integrating different communities of users into joint discussions and interactions at the same location. This working mode greatly promotes the synergy pertaining to data analysis and visualization in IPT systems.

In future work, the first priority will be bringing more informative visualizations into the data pipeline, for instance, getting more categories and more formats of volumetric visualizations that would give users a better comprehension of the process output. Furthermore, involving constructive usability and usage testing is another critical concern, for example, designing a systematic user study by engaging adequate people to perform specific tasks in our AR system, collecting feedback, and revising the system correspondingly. In addition, prototyping on advanced AR equipment such as Microsoft HoloLens 2 or Magic Leap headsets will be another concentration, which should break the limit in screen displaying and yield higher interactivity and immersivity for targeted users.

## Data Availability

The data presented in this study are available on request from the corresponding authors.
